# Myelin oligodendrocyte glycoprotein antibody-associated isolated aseptic meningitis: a single−center pediatric case series

**DOI:** 10.3389/fimmu.2025.1699683

**Published:** 2025-10-23

**Authors:** Kang Liu, Suzhen Sun, Fang Chen, Wenjuan Wu, Xin Li, Lingyu Pang, Wei Wang

**Affiliations:** ^1^ First Department of Neurology, Hebei Children’s Hospital, Shijiazhuang, China; ^2^ First Department of Neurology, Hebei Clinical Medicine Research Center for Children’s Health and Diseases, Shijiazhuang, China; ^3^ First Department of Neurology, Hebei Provincial Key Laboratory for Pediatric Epilepsy and Neurological Disorders, Shijiazhuang, China

**Keywords:** isolated aseptic meningitis, myelin oligodendrocyte glycoprotein antibody, prolonged fever, leptomeningeal enhancement, children

## Abstract

**Background and purpose:**

Aseptic meningitis represents a rare and underrecognized phenotype of myelin oligodendrocyte glycoprotein (MOG) antibody-associated disease (MOGAD). Despite sporadic case reports, comprehensive series studies remain scarce. This study aimed to describe the clinical characteristics of MOG antibody-associated aseptic meningitis (MOGAM) in pediatric patients without neuroparenchymal lesions.

**Methods:**

We reviewed the medical records of pediatric patients from January 2019 to July 2025, focusing on cases diagnosed with MOGAM in the absence of neuroparenchymal lesions. Clinical manifestations, brain magnetic resonance imaging (MRI) findings, laboratory results, treatment regimens, and clinical outcomes were retrospectively analyzed.

**Results:**

Among 159 children with seropositive MOG antibodies, 11 (6.9%) met the inclusion criteria. The mean age at disease onset was 8.3 years (8.3 ± 3.2 years), with a male-to-female ratio of 5:6. The most common symptoms were fever (10/11), lasting from 3 to 50 days (median, 13 days; interquartile range [IQR], 8.0–22.7 days), and headache (10/11). Meningeal irritation signs were positive in 4 patients (4/11). Nine patients had peripheral blood leukocytosis (21.6 ± 3.6×10^9^/L; range, 17.2–29.1×10^9^/L), and 10 had elevated neutrophil-to-lymphocyte ratio (NLR; median, 4.5; IQR, 4.0–7.9; range, 3.0–14.5) as well as erythrocyte sedimentation rate (ESR; 38.4 ± 10.7 mm/h; range, 25–62 mm/h). All patients had cerebrospinal fluid (CSF) pleocytosis (117.4 ± 62.0/μL; range, 50–201/µL); 2 had elevated CSF pressure (range, 250–350 mmH_2_O); 4 had slightly increased CSF protein levels (range, 0.48–0.96 g/L); and none tested positive for infectious pathogens. Six patients showed abnormal electroencephalogram (EEG) results, including focal interictal epileptiform discharges or slow waves (3/6) and slow background activities (5/6). Brain MRI showed linear hyperintense signals along the bilateral cerebral sulci on fluid-attenuated inversion recovery (FLAIR) sequences in 4 patients (4/11), while no abnormalities were observed in 7 patients (7/11). Leptomeningeal enhancement (LME) was detected in 2 of 3 patients who underwent contrast-enhanced brain MRI. All patients received immunotherapy, with 2 administered immunosuppressive therapy after relapse. At the last follow-up, all patients achieved favorable clinical outcomes.

**Conclusion:**

Myelin oligodendrocyte glycoprotein antibody-associated isolated aseptic meningitis is a novel and underrecognized clinical phenotype. For pediatric patients presenting with prolonged or recurrent fever and headache, particularly those with CSF pleocytosis, leptomeningeal enhancement (LME), and poor response to anti-infective therapy, early testing for MOG-IgG is strongly recommended. Timely identification of this distinct phenotype may facilitate early diagnosis, initiation of effective immunotherapy, and better clinical outcomes.

## Introduction

Myelin oligodendrocyte glycoprotein antibody-associated disease (MOGAD) is an emerging, distinct immune-mediated inflammatory disorder that primarily affects the central nervous system (CNS). With the continuous advancement and widespread application of detection assays for myelin oligodendrocyte glycoprotein (MOG) antibodies, MOG antibodies (MOG-Abs) are increasingly being identified in a broadened spectrum of acquired demyelinating disorders. MOG-Abs are particularly prevalent in the pediatric population, with diverse onset ages and growing phenotypic heterogeneity (1, 2). In addition to being involved in central nervous system demyelinating disorders—including acute disseminated encephalomyelitis (ADEM), optic neuritis (ON), myelitis, and neuromyelitis optica spectrum disorder (NMOSD)—MOG-Abs have also been identified as the most prevalent autoantibodies in autoimmune encephalitis (AE). Furthermore, the appearance of rare phenotypic variants, such as cortical encephalitis, brainstem encephalitis, AE with normal brain magnetic resonance imaging (MRI), aseptic meningitis, and isolated seizures, suggests that MOG-Abs may exert a broader pathogenic role in immune-mediated inflammation beyond demyelination. Notably, the first detection of MOG-Abs in combined central and peripheral demyelination (CCPD) syndromes in 2018 (3) has spurred investigations into peripheral nerve involvement related to MOG antibodies. These findings highlight that the understanding of MOGAD remains in a state of dynamic evolution, characterized by increasing clinical complexity and expanding phenotypic manifestations.

Recently, sporadic cases of aseptic meningitis associated with MOG-Abs have been reported. Given that its clinical manifestations closely mimic those of infectious meningitis—including fever, headache, and cerebrospinal fluid (CSF) leukocytosis—and its lack of disease-specific features, this rare phenotypic variant of MOGAD is highly prone to misdiagnosis. However, negative results from etiological tests, lack of response to anti-infective therapy, and—conversely—positive response to immunotherapy have directed attention toward its immune-mediated etiology, particularly the involvement of MOG-Abs. This condition has therefore been gradually recognized as MOG antibody-associated aseptic meningitis (MOGAM) (6) and integrated into the phenotypic spectrum of MOGAD. Cases involving prolonged fever (4) or chronic headache (5) have also been documented. It is important to note that MOGAM may present either in isolation or as initial manifestations that precede, coincide with, or follow demyelinating events; additionally, there have even been reports describing subclinical demyelinating events—identifiable solely through neuroimaging abnormalities (7).

Given its rarity, reports on MOGAM remain scarce, especially when it presents as an isolated phenotype in the pediatric population. In this study, we evaluated eleven pediatric patients with isolated MOGAM, who showed no clinical symptoms or neuroimaging evidence of neuroparenchymal lesions, at a single center in Northern China. The aim of this study was to characterize the clinical profile of pediatric isolated MOGAM and to refine clinical management strategies for this distinct, underrecognized phenotype.

## Patients and methods

### Participants

This was a retrospective single-center cohort study. We reviewed the clinical data of patients hospitalized at Hebei Children’s Hospital from January 2019 to July 2025. Patients were enrolled in accordance with the following criteria: (i) Diagnosis of aseptic meningitis, confirmed by the combination of clinical symptoms, physical signs, and CSF test results; (ii) Age ≤ 168 months (14 years old); (iii) Positive serum myelin oligodendrocyte glycoprotein immunoglobulin G (MOG-IgG), with negative aquaporin-4 immunoglobulin G (AQP4-IgG) and negative other autoimmune antibodies in both serum and CSF (including NMDAR, AMPA1, AMPA2, GABAB, LGI1, CASPR2, GAD65, mGluR5, GFAP, as well as Hu, Yo, Ri, Ma2, CV2, Amphiphysin, ANNA-3, Tr, PCA-2, and GAD antibodies); (iv) Absence of encephalopathy, focal neurological deficits, and neuroparenchymal lesions on magnetic resonance imaging (MRI); and (v) Exclusion of aseptic meningitis caused by other etiologies, including systemic lupus erythematosus (SLE), rheumatoid arthritis, Kawasaki disease (KD), Behçet’s disease, and other autoimmune or inflammatory conditions.

### Clinical data collection

The clinical dataset included the following essential variables: age, gender, duration of hospitalization, clinical symptoms, laboratory parameters, brain MRI findings, treatment regimens, and clinical outcomes. All brain MRI scans were acquired using a 3-tesla (3T) magnetic field strength. Following hospital discharge, all patients were followed up through outpatient clinic visits.

### Assays for MOG antibodies

Serum MOG antibodies were detected using live cell-based assays (live CBAs), while MOG antibodies in cerebrospinal fluid (CSF) were detected via fixed cell-based assays (fixed CBAs). All assays were performed according to the standardized operating procedures. Based on the laboratory’s validated assay parameters, MOG antibody titers were stratified as follows: ≥ 1:100 was defined as high-positive, 1:10 to 1:32 as low-positive, and < 1:10 as negative.

### Ethical approval and informed consent

This study received approval from the Medical Ethics Committee of Hebei Children’s Hospital, affiliated with Hebei Medical University. Written informed consent was obtained from the parents or legal guardians of all participating pediatric patients.

## Results

### Demographics and clinical data

Among the 159 pediatric patients with serum MOG antibody positivity reviewed during the study period, 11 (11/159, 6.9%) met the aforementioned inclusion criteria. Their demographic data and clinical characteristics are detailed in **Table 1**. The mean age at disease onset was 8.3 years (8.3 ± 3.2 years; range, 4–14 years), with a male-to-female ratio of 5:6. None of the 11 patients had a prior history of CNS disease or autoimmune disease.

**Table 1:** Detailed demographic and clinical characteristics of 11 patients with isolated MOGAM.

Variables	No./Total No. (%)
Age at onset (mean ± SD), year	8.3 ± 3.2
Female	6/11 (54.5%)
Clinical Characteristics	
Fever	10/11 (90.9%)
The duration of fever (IQR), day	13 (8.0–22.7)
Headache	10/11 (90.9%)
Vomiting	6/11 (54.5%)
Seizure	2/11 (18.2%)
Meningeal irritation sign	4/11 (36.4%)
Laboratory findings	
Blood	
Leukocytosis	9/11 (81.8%)
Elevated NLR	10/11 (90.9%)
Elevated ESR	10/11 (90.9%)
Elevated CRP	4/11 (36.4%)
Elevated PCT	4/11 (36.4%)
CSF	
ICP>200mmH_2_O	2/8 (25.0%)
Leukocytosis, >10 cells/μL	11/11 (100%)
Elevated protein, >0.45 g/L	4/11 (36.4%)
OCB	4/11 (36.4%)
MOG-IgG in Serum	11/11 (100%)
MOG-IgG in CSF	3/11 (27.3%)
EEG findings	
Slow background activity	5/11 (45.5%)
Focal interictal epileptiform discharges or slow waves	3/11 (27.3%)
MRI findings	
Linear hyperintense signals along cerebral sulci on FLAIR sequence	4/11 (36.4%)
Negative MRI	7/11 (63.6%)
Leptomeningeal enhancement	2/3 (66.7%)
Time from onset to diagnosis (IQR), day	17 (11–28)
Treatment	
Antibiotics used	7/11 (63.6%)
Steroid	11/11 (100%)
IVIG	10/11 (90.9%)
Immunosuppressive drugs	2/11 (18.2%)
Clinical outcomes	
Duration of follow-up (mean ± SD), month	12.6 ± 11.5
Relapse	2/11 (18.2%)

CSF, cerebrospinal fluid; CRP, C-reactive protein; EEG, electroencephalogram; ESR, erythrocyte sedimentation rate; FLAIR, fluid-attenuated inversion recovery; ICP, intracranial pressure; IQR, interquartile range; IVIG, intravenous immunoglobulin; MOG-IgG, myelin oligodendrocyte glycoprotein immunoglobulin G; MOGAM, myelin oligodendrocyte glycoprotein-associated aseptic meningitis; MRI, magnetic resonance imaging; NLR, neutrophil-lymphocyte ratio; No., number; OCB, oligoclonal band; PCT, procalcitonin; SD, standard deviation; WBC, white blood cell.

The clinical symptoms included fever (10/11, 90.9%), with a duration from 3 to 50 days (median, 13 days; interquartile range [IQR], 8.0–22.7 days), headache (10/11, 90.9%), vomiting (6/11, 54.5%), and seizures (2/11, 18.2%), with fever and headache being the most common. The majority of fevers (8/10, 80%) lasted longer than one week, with 3 cases (3/10, 30%) persisting for over three weeks. Additionally, meningeal irritation signs—predominantly neck stiffness—were positive in four patients (4/11, 36.4%; age range, 8–14 years). Importantly, none of the patients displayed psychiatric or behavioral abnormalities, movement disorders, sleep disturbances, dysautonomia, visual impairment, or spinal cord involvement.

### Laboratory findings

All patients underwent a complete blood count (CBC). Among these, 9 (9/11, 81.8%) had leukocytosis, with a mean leukocyte count of 21.6×10^9^/L (21.6 ± 3.6×10^9^/L; range, 17.2–29.1×10^9^/L; reference range: 4–10×10^9^/L). Additionally, 10 patients (10/11, 90.9%) exhibited a significantly elevated neutrophil-to-lymphocyte ratio (NLR; median, 4.5; IQR, 4.0–7.9; range, 3.0–14.5). Systemic inflammatory markers, including erythrocyte sedimentation rate (ESR), C-reactive protein (CRP), and procalcitonin (PCT), were measured in all 11 patients: 10 (10/11, 90.9%) had elevated ESR (38.4 ± 10.7 mm/h; range, 25–62 mm/h; reference range, 0–10 mm/h); 4 (4/11, 36.4%) had mildly increased CRP levels (range, 8.4–14.9 mg/L; reference range, 0–8 mg/L); and 4 (4/11, 36.4%) had slightly elevated PCT levels (range, 0.10–0.15 μg/L; reference range, 0–0.06 μg/L).

All patients (11/11, 100%) exhibited CSF pleocytosis (117.4 ± 62.0/μL; range, 50–201/μL; reference range, 0–10/μL), predominantly consisting of mononuclear leukocytes. CSF pressure was measured in 8 patients, 2 of whom had elevated pressure (range, 250–350 mmH_2_O; reference range, 80–180 mmH_2_O). Four patients (4/11, 36.4%) showed slightly elevated CSF protein levels (range, 0.48–0.96 g/L; reference range, 0.28–0.45 g/L), whereas none had abnormal CSF glucose levels (reference range, 2.5–4.4 mmol/L). CSF bacterial cultures and polymerase chain reaction (PCR) assays targeting herpes simplex virus (HSV), Epstein-Barr virus (EBV), cytomegalovirus (CMV), and Mycoplasma pneumoniae (MP) were negative in all patients. Furthermore, metagenomic next-generation sequencing (mNGS) was performed on CSF samples from 6 patients, with no infectious pathogens detected.

Serum MOG-IgG was positive in all patients, whereas CSF MOG-IgG was positive in only 3 cases. The titers of serum MOG-IgG ranged from 1:10 to 1:3200, and those of CSF MOG-IgG ranged from 1:10 to 1:32. Additionally, CSF-specific oligoclonal bands (OCBs) were detected in 4 patients (4/11, 36.4%).

### Electroencephalogram findings

Electroencephalogram (EEG) was conducted in all patients, with 6 cases (6/11, 54.5%) showing abnormal results. Specifically, 3 patients presented with focal interictal epileptiform discharges or slow waves, and 5 patients exhibited diffuse slow background activity. During the EEG monitoring period, no clinical seizures or subclinical status epilepticus were recorded.

### MRI findings

**Figure 1:**
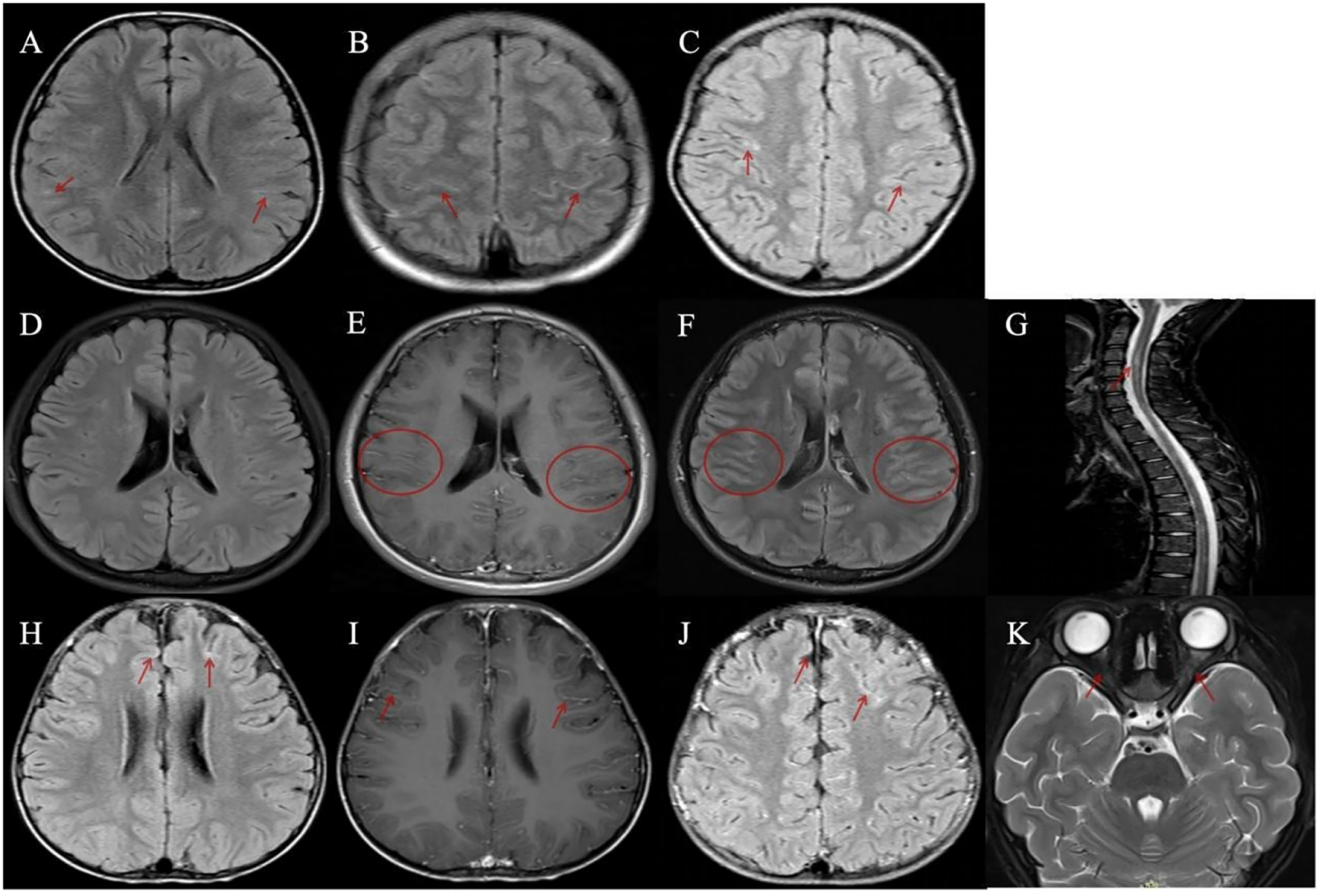
Magnetic resonance imaging (MRI) findings in patients with meningeal abnormalities and contrast enhancement. **(A–C, H)** Four patients demonstrated linear hyperintense signals along the cerebral sulci on fluid-attenuated inversion recovery (FLAIR) sequences. **(D–K)** Two patients with disease relapse exhibited leptomeningeal enhancement. **(D–G)** A patient with recurrent myelitis: **(D)** FLAIR imaging showed no abnormal lesions; **(E, F)** Bilateral multiple leptomeningeal enhancements were observed on gadolinium-enhanced T1-weighted axial and FLAIR images; **(G)** Spinal MRI revealed multiple abnormal signals involving the cervical and thoracic spinal segments. **(H–K)** A patient with recurrent optic neuritis: **(H)** A representative image showed mild linear hyperintense signals along the cerebral sulci on FLAIR sequence; **(I, J)** Bilateral leptomeningeal enhancements were observed on gadolinium-enhanced T1-weighted axial and FLAIR images; **(K)** Orbital MRI demonstrated bilateral optic nerve swelling with abnormal signals.

Brain MRI was performed in all 11 patients, with detailed findings summarized in **Table 1**. Among these patients, 4 (4/11, 36.4%) exhibited linear hyperintense signals along segments of the bilateral cerebral sulci on fluid-attenuated inversion recovery (FLAIR) sequences (**Figures 1A–C, H**), while 7 (7/11, 63.6%) showed no abnormal signals (**Figure 1D**). Gadolinium-enhanced brain MRI was performed in only 3 patients, with 2 showing leptomeningeal enhancement (LME) (**Figures 1E, F, I, J**) and 1 showing no contrast enhancement. Importantly, none of the 11 patients had radiological evidence of involvement in the cerebral cortex, subcortical white matter, or deep gray matter. Furthermore, orbital and spinal cord MRI were performed in all 11 patients, with no lesions detected.

### Treatment and outcomes

Details regarding the treatment regimens and clinical outcomes of the 11 patients are outlined in **Table 1**. Given the initial suspicion of intracranial infection, empirical anti-infective therapy was administered to all 11 patients. Considering the potential risk of herpes simplex encephalitis (HSE), all patients received intravenous acyclovir, with 7 cases additionally receiving empirical antibiotic therapy. None of the patients, however, demonstrated a favorable clinical response to the anti-infective therapy.

Following the definitive diagnosis of MOGAM, immunotherapy was promptly initiated in all pediatric patients. The median latency period from symptom onset to definitive diagnosis and subsequent initiation of immunotherapy was 17 days (IQR, 11–28 days; range, 10–54 days). At our center, all 11 patients received high-dose intravenous methylprednisolone (IVMP; 20 mg/kg/day for 3 days, followed by a taper to 10 mg/kg/day for 3 days, then a further reduction to 5 mg/kg/day for 3 days, and a final taper to 2.5 mg/kg/day for 3 days before discontinuation), subsequently transitioning to oral prednisone (OP; 1.5–2.0 mg/kg/day, tapered over 3–6 months). Among them, 10 patients additionally received intravenous immunoglobulin (IVIG; 1 g/kg/day for 2 days) in combination, with one case declining IVIG due to economic constraints. During the first acute episode, no patients received immunosuppressive therapy.

The median duration of hospital stay was 21 days (IQR, 19–26 days; range, 15–40 days). Notably, immunotherapy resulted in rapid and significant remission of clinical symptoms, and all patients achieved favorable clinical outcomes at discharge.

### Follow-up

After discharge, all 11 patients attended outpatient follow-up. With a mean follow-up duration of 12.6 months (12.6 ± 11.5 months; range, 1–37 months), the majority of patients (9/11, 81.8%) exhibited a monophasic disease course, whereas 2 (2/11, 18.2%) experienced recurrent demyelinating events, with the time intervals from initial episode to relapse being 10 months and 15 months, respectively.

One patient developed acute myelitis, manifesting as bilateral lower limb weakness and urinary retention. Spinal MRI revealed multiple abnormal signals in the cervical and thoracic segments of the spinal cord (**Figure 1G**), whereas electromyography (EMG) and cranial MRI showed no abnormalities. Notably, the serum MOG-IgG remained persistently positive at 8 months post-discharge. Another patient presented with bilateral optic neuritis, with clinical manifestations of bilateral blurred vision. Visual evoked potential (VEP) showed prolonged latency and reduced amplitude of the P100 wave. Orbital MRI indicated bilateral optic nerve thickening with abnormal signals (**Figure 1K**), while brain MRI was normal. This case also had persistently positive serum MOG-IgG, which was confirmed at 16 months post-discharge.

Following hormone therapy, immunosuppressive therapy was initiated in the two patients with disease relapse—one received tocilizumab (TCZ) and the other rituximab (RTX). At the time of the last follow-up, all patients had achieved favorable clinical outcomes. Specifically, clinical symptoms were relieved, and the abnormal findings on MRI either returned to normal or showed marked improvement.

## Discussion

In this study, we evaluated the clinical characteristics of 11 pediatric patients with aseptic meningitis, positive serum MOG-IgG, and no evidence of neuroparenchymal lesions at the initial episode. To our knowledge, this is the first case series focusing on Asian children that specifically describes isolated MOG antibody-associated aseptic meningitis (MOGAM)—a rare and underrecognized phenotype of MOGAD.

In 1999 (8), experimental autoimmune meningitis (EAM), a distinct model of immune-mediated aseptic meningitis, was first induced in CD28-deficient (CD28^-^/^-^) C57BL/6 mice following immunization with MOG. This finding suggested that specific costimulatory factors may drive the differential development, progression, and phenotypic manifestation of MOGAD. However, MOG antibody-associated aseptic meningitis (MOGAM) remained undocumented until 2019, when the first adult case was reported (9), followed by the first pediatric case in 2020 (10). With a growing number of cases being reported, emerging evidence—including shared neuroimaging patterns, clinical manifestations, CSF parameters, and treatment responses—indicates that MOGAM, along with FLAMES (FLAIR hyperintense cortical lesions in myelin oligodendrocyte glycoprotein-associated encephalitis with seizures), UCCE (unilateral cerebral cortical encephalitis), BFCCE (bilateral medial frontal cerebral cortical encephalitis), BPCLI (bilateral parafalcine cortical and leptomeningeal impairment), and FUEL (FLAIR-variable unilateral enhancement of the leptomeninges)—lies on a continuum of meningo-cortical manifestations in MOGAD (11).

In this study, the incidence of isolated MOGAM was 6.9%, which is significantly lower than the 17.6% reported in a previous retrospective study (12). This discrepancy may be primarily attributed to differences in study populations, specifically that the prior study had a broader inclusion criteria, encompassing adult cases, cases of non-isolated aseptic meningitis, and those with subclinical demyelinating lesions. Given the overlapping clinical and laboratory features between isolated MOGAM and infectious meningoencephalitis, aseptic meningitis is seldom considered as an initial diagnosis. Furthermore, the lack of early MOG-IgG testing at disease onset may lead to missed diagnoses, potentially contributing to the underestimation of its true incidence (13). A recent study (14) has identified MOG-encephalitis as the most prevalent subtype of autoimmune encephalitis in children. Nevertheless, whether MOG antibodies represent the leading cause of autoimmune aseptic meningitis in the pediatric population still warrants further investigation.

This study revealed no obvious gender predominance (male-to-female ratio, 1:1.2), with 9 of 11 children (81.8%) being over 6 years old. These findings are consistent with those reported in Gu’s study (12) but contrast with Lin’s study (6), which documented a male predominance (75%) and a younger age distribution, with 5 of 8 children (62.5%) under 6 years old. Additional findings regarding isolated MOGAM in this study included the following: nonspecific clinical manifestations (predominantly fever and headache), peripheral blood leukocytosis, CSF pleocytosis, elevated ESRs, negative pathogen detection results, FLAIR hyperintense signals along the cerebral sulci or leptomeningeal enhancement (LME) on brain MRI, ineffective anti-infective therapy, and significant clinical improvement following immunotherapy. Owing to the nonspecific clinical manifestations and auxiliary examination findings, isolated aseptic meningitis is frequently misdiagnosed as an infectious disease, particularly when it presents in isolation. More importantly, delayed initiation of immunotherapy may lead to nearly half of patients with MOGAM (46.9%) developing new focal neurological deficits (12). However, given the increasing number of reported pediatric cases (4, 5, 10, 15–17), the following clinical features should raise suspicion for MOGAM and prompt MOG-IgG testing: (1) prolonged fever or persistent headache, particularly when accompanied by elevated intracranial pressure (ICP), CSF pleocytosis, leptomeningeal enhancement, and no response to anti-infective therapy; (2) peripheral blood leukocytosis in most patients, with over half having white blood cell (WBC) counts exceeding 20×10^9^/L—a finding rarely observed in other MOGAD subtypes or autoimmune encephalitis; (3) normal CSF glucose levels and no significant elevations in systemic inflammatory markers (e.g., CRP and PCT), which facilitates differentiation of MOGAM from bacterial meningitis. Notably, the first documented case of a pediatric patient with isolated MOGAM—who achieved spontaneous remission and remained symptom-free during an extended follow-up period—has been reported (16). This observation raises the question of whether more patients with isolated MOGAM might have been overlooked or misdiagnosed as infectious diseases, particularly in instances where symptoms resolved spontaneously without the administration of immunotherapy.

Currently, controversy persists regarding the specific biomarkers for the differential diagnosis and relapse prediction of MOGAD. First, with advances in neuroimaging technologies and the deepening of related research, leptomeningeal enhancement (LME) has emerged as a novel and relatively specific early neuroradiological marker for pediatric MOGAD. However, in the present study, only three children underwent contrast-enhanced MRI due to issues with patient compliance and economic constraints, and LME was detected in two cases. Notably, the reported incidence of LME in MOGAD cohorts varies across populations: 6% in adults (18), 33% in children (19), and 46% in a mixed pediatric-adult cohort (20). In a pediatric study (21), LME was observed exclusively in MOGAD cases and was absent in pediatric-onset multiple sclerosis (POMS) or aquaporin-4 antibody-positive neuromyelitis optica spectrum disorder (AQP4-NMOSD). Compared with MOGAD patients without LME, those with LME exhibited a higher incidence of fever, headache, and seizures; however, the association between LME and disease relapse in MOGAD remains debated. Notably, LME predominantly appears in the early stages of the disease, with its incidence declining over time and persistent LME being rare. Second, the neutrophil-to-lymphocyte ratio (NLR), defined as the ratio of peripheral blood neutrophil count to lymphocyte count, has been found to be elevated during acute MOGAD attacks. It may facilitate the early identification of disease relapse and enable the prediction of a relapsing course at disease onset. Furthermore, elevated NLR levels have been associated with fever and isolated optic neuritis (ON)—a relatively milder phenotype of MOGAD (22). In our cohort, most patients with isolated MOGAM (10/11, 90.9%) exhibited a significant increase in NLR, with the highest NLR recorded in two patients (14.5 and 12.9) experiencing disease relapse. Therefore, further studies are required to confirm whether NLR can serve as a clinically valuable biomarker for MOGAD. It also remains to be determined whether MOGAD patients with elevated NLR levels could benefit from a more gradual hormone tapering schedule.

Various phenotypes of MOGAD may appear either in isolation or concurrently across different disease stages, with aseptic meningitis typically presenting in the early phase. Recent studies (6, 12) have demonstrated that over half (58%) of patients initially diagnosed with MOGAM progressed to develop other phenotypic manifestations of MOGAD. Additionally, the time interval from disease onset to the initiation of immunotherapy was longer in the progressive group than in the isolated group (17–32 days vs. 13–24 days), suggesting a potential association between delayed immunotherapy and disease progression in MOGAM. However, several observations deserve attention: (a) The difference in the aforementioned time interval between the progressive group and isolated group did not reach statistical significance; (b) In the present study, this time interval varied from 10 to 54 days, yet none of the patients developed other MOGAD phenotypes; (c) One reported case of pediatric isolated MOGAM achieved spontaneous remission without immunotherapy (16). Therefore, further investigation into the risk factors associated with the progression of MOGAM is warranted.

Consistent with other phenotypes of MOGAD, patients with isolated MOGAM generally respond well to immunotherapy, and most achieve favorable clinical outcomes. However, as a newly recognized and rare phenotype, the factors contributing to relapse in MOGAM remain poorly elucidated. In this study, the relapse rate was 18.2%, which falls within the range (16.7%–25%) reported in previous research. By categorizing MOGAM patients into three groups based on clinical and neuroimaging features, Gu et al. (12) found that the complex type—defined as monophasic AM with subclinical demyelination on MRI—had a significantly higher relapse rate (50.0%) than the progressive type (AM progressing to develop other neurological symptoms, 20.0%) and the simple type (monophasic AM alone, 8.3%). In addition, Lin et al. (6) indicated that inadequate steroid treatment and persistent MOG-IgG positivity may be associated with disease relapse. While among the 9 non-relapsing patients in the present study, 5 had discontinued steroid therapy, and 6 remained persistently MOG-IgG positive. Therefore, the causal roles of inadequate steroid treatment and persistent MOG-IgG positivity in disease recurrence remain unclear. Notably, subclinical demyelination lesions in MOGAM indeed warrants heightened clinical attention and close long-term monitoring.

Although MOGAD typically involves demyelination, increasing clinical evidence has revealed non-demyelination-associated phenotypes and MRI-negative findings. Currently, the role of anti-MOG antibodies in the development of aseptic meningitis remains poorly understood, and the potential mechanisms include: 1) The meninges maintain a relatively stable immune microenvironment under physiological conditions, with tightly regulated interactions between resident immune cells and the blood-brain barrier (BBB) (23). Upon crossing the BBB, anti-MOG antibodies may facilitate the extravasation of peripheral immune cells and pro-inflammatory mediators into the meningeal space. Furthermore, anti-MOG antibodies can interact with resident immune cells, stimulating them to secrete cytokines and chemokines. This process further triggers an inflammatory cascade, disrupts immune homeostasis, and ultimately leads to meningitis. 2) MOG can induce experimental autoimmune meningitis in CD28-deficient (CD28^−^/^−^) C57BL/6 mice. Notably, the CD28 co-stimulatory signal is crucial for the survival and functional integrity of regulatory T cells (Tregs) (24). Therefore, analyzing Treg profiles may provide valuable insights into the underlying pathogenic mechanisms. 3) The anti-MOG antibody itself may not be directly associated with meningitis. Instead, other unidentified autoimmune antibodies may coexist with anti-MOG antibodies and exert a synergistic effect, thereby contributing to the pathogenesis of this disease.

This study had several limitations. First, as a single-center retrospective study with a small sample size, the generalizability of the findings may be constrained. Second, contrast-enhanced MRI was performed only in a subset of cases, thereby restricting a comprehensive evaluation of LME in isolated MOGAM. Finally, the follow-up duration was relatively short in some cases, which may have precluded the capture of long-term outcomes. Therefore, prospective multicenter studies involving larger cohorts, more comprehensive clinical data, and extended follow-up periods are required to better delineate the clinical characteristics and long-term prognosis of isolated MOGAM.

## Conclusion

Isolated MOG antibody-associated aseptic meningitis (MOGAM), defined as MOGAM without neuroparenchymal lesions, is a rare and underrecognized phenotype. Clinically, in pediatric patients presenting with prolonged fever or headache, especially those unresponsive to anti-infective therapy, even in the absence of neuroparenchymal lesions on brain MRI, serum MOG antibody testing should be performed as early as possible.

Our study demonstrates that pediatric patients with isolated MOGAM generally achieve favorable outcomes following immunotherapy. However, the factors associated with disease relapse or progression to other phenotypic manifestations of MOGAD remain unclear. Future studies involving larger, multicenter cohorts are essential to identify factors influencing the progression, recurrence, and long-term prognosis of MOGAM.

The study was approved by the Medical Ethics Committee of Hebei Children’s Hospital, affiliated with Hebei Medical University. Informed consent was obtained from the parents or legal guardians of all participating children.

## Data Availability

The raw data supporting the conclusions of this article will be made available by the authors, without undue reservation.

## References

[1] Reindl M Waters P. Myelin oligodendrocyte glycoprotein antibodies in neurological disease. Nat Rev Neurol. (2019) 15:89–102. doi: 10.1038/s41582-018-0112-x, PMID: 30559466

[2] de Mol CL Wong Y van Pelt ED Wokke B Siepman T Neuteboom RF. The clinical spectrum and incidence of anti-MOG-associated acquired demyelinating syndromes in children and adults. Mult Scler. (2020) 26:806–14. doi: 10.1177/1352458519845112, PMID: 31094288 PMC7294530

[3] Vazquez Do Campo R Stephens A Marin Collazo IV Rubin DI. MOG antibodies in combined central and peripheral demyelination syndromes. Neurol Neuroimmunol Neuroinflamm. (2018) 5:e503. doi: 10.1212/NXI.0000000000000503, PMID: 30246057 PMC6147156

[4] Udani V Badheka R Desai N. Prolonged fever: an atypical presentation in MOG antibody-associated disorders. Pediatr Neurol. (2021) 122:1–6. doi: 10.1016/j.pediatrneurol.2021.03.006, PMID: 34198219

[5] Hadjiev J McCarthy J Madion L Mondok L. Six year old with chronic headache: an unexpected meningitis mimic. WMJ. (2024) 123:138–40., PMID: 38718245

[6] Lin S Long W Wen J Su Q Liao J Hu Z. Myelin oligodendrocyte glycoprotein antibody-associated aseptic meningitis without neurological parenchymal lesions: A novel phenotype. Mult Scler Relat Disord. (2022) 68:104126. doi: 10.1016/j.msard.2022.104126, PMID: 36115288

[7] Nagabushana D Shah R Pendharkar H Agrawal A Kulkarni GB Rajendran S. MOG antibody seropositive aseptic meningitis: A new clinical phenotype. J Neuroimmunol. (2019) 333:476960. doi: 10.1016/j.jneuroim.2019.05.001, PMID: 31108402

[8] Perrin PJ Lavi E Rumbley CA Zekavat SA Phillips SM. Experimental autoimmune meningitis: a novel neurological disease in CD28-deficient mice. Clin Immunol. (1999) 91:41–9. doi: 10.1006/clim.1998.4684, PMID: 10219253

[9] Narayan RN Wang C Sguigna P Husari K Greenberg B. Atypical Anti-MOG syndrome with aseptic meningoencephalitis and pseudotumor cerebri-like presentations. Mult Scler Relat Disord. (2019) 27:30–3. doi: 10.1016/j.msard.2018.10.003, PMID: 30300850

[10] Leinert J Neumaier-Probst E Kutschke G Tenenbaum T. MOG antibody associated demyelinating syndrome presenting as aseptic meningitis in a 6-year-old boy. Mult Scler Relat Disord. (2020) 41:102050. doi: 10.1016/j.msard.2020.102050, PMID: 32200343

[11] Budhram A Mirian A Sharma M. Meningo-cortical manifestations of myelin oligodendrocyte glycoprotein antibody-associated disease: Review of a novel clinico-radiographic spectrum. Front Neurol. (2022) 13:1044642. doi: 10.3389/fneur.2022.1044642, PMID: 36341089 PMC9630470

[12] Gu M Mo X Fang Z Zhang H Lu W Shen X. Characteristics of aseptic meningitis-like attack-an underestimated phenotype of myelin oligodendrocyte glycoprotein antibody-associated disease. Mult Scler Relat Disord. (2023) 78:104939. doi: 10.1016/j.msard.2023.104939, PMID: 37611382

[13] Forcadela M Rocchi C San Martin D Gibbons EL Wells D Woodhall MR. Timing of MOG-igG testing is key to 2023 MOGAD diagnostic criteria. Neurol Neuroimmunol Neuroinflamm. (2023) 11:e200183. doi: 10.1212/NXI.0000000000200183, PMID: 37977848 PMC10758949

[14] Fellmeth RH Kousoulos L Korenke GC Christen HJ Monazahian M Dargvainiene J. MOG-encephalitis is the most prevalent autoimmune encephalitis in children: MERIN study data on encephalitis. Neuropediatrics. (2025) 56:226–33. doi: 10.1055/a-2579-6247, PMID: 40209801

[15] Vibha D Singh RK Salunkhe M Dash D Tripathi M. MOG antibody syndrome presenting as aseptic meningitis: an evolving spectrum. Neurol Sci. (2021) 42:321–3. doi: 10.1007/s10072-020-04558-4, PMID: 32632636

[16] Hino-Fukuyo N Kawai E Itoh S Oba S Sato Y Abe S. Myelin oligodendrocyte glycoprotein antibody-associated disease in a patient with symptoms of aseptic meningitis who achieved spontaneous remission: A case report and review of the literature. Brain Dev. (2023) 45:456–61. doi: 10.1016/j.braindev.2023.05.002, PMID: 37246116

[17] Wong WK Troedson C Peacock K Brilot-Turville F Menezes MP Dale RC. Steroid-responsive aseptic meningitis with raised intracranial pressure syndrome associated with myelin oligodendrocyte glycoprotein autoantibodies. J Paediatr Child Health. (2022) 58:2322–6. doi: 10.1111/jpc.16189, PMID: 36000565 PMC10087128

[18] Cobo-Calvo A Ruiz A Maillart E Audoin B Zephir H Bourre B. Clinical spectrum and prognostic value of CNS MOG autoimmunity in adults: The MOGADOR study. Neurology. (2018) 90:e1858–69. doi: 10.1212/WNL.0000000000005560, PMID: 29695592

[19] Gadde JA Wolf DS Keller S Gombolay GY. Rate of leptomeningeal enhancement in pediatric myelin oligodendrocyte glycoprotein antibody-associated encephalomyelitis. J Child Neurol. (2021) 36:1042–6. doi: 10.1177/08830738211025867, PMID: 34547933 PMC9054459

[20] Elsbernd P Cacciaguerra L Krecke KN Chen JJ Gritsch D Lopez-Chiriboga AS. Cerebral enhancement in MOG antibody-associated disease. J Neurol Neurosurg Psychiatry. (2023) 95:14–8. doi: 10.1136/jnnp-2023-331137, PMID: 37221051 PMC10679850

[21] Goldman-Yassen A Lee A Gombolay G. Leptomeningeal enhancement in pediatric anti-myelin oligodendrocyte glycoprotein antibody disease, multiple sclerosis, and neuromyelitis optica spectrum disorder. Pediatr Neurol. (2024) 153:125–30. doi: 10.1016/j.pediatrneurol.2024.01.026, PMID: 38382244 PMC10940200

[22] Kim YH Kim SY Yoo IH Lim BC Chae JH Kim KJ. Clinical utility of complete blood count indices in pediatric MOG antibody-associated disease. Mult Scler Relat Disord. (2025) 98:106446. doi: 10.1016/j.msard.2025.106446, PMID: 40253903

[23] Castellani G Croese T Peralta Ramos JM Schwartz M. Transforming the understanding of brain immunity. Science. (2023) 380:eabo7649. doi: 10.1126/science.abo7649, PMID: 37023203

[24] Bour-Jordan H Esensten JH Martinez-Llordella M Penaranda C Stumpf M Bluestone JA. Intrinsic and extrinsic control of peripheral T-cell tolerance by costimulatory molecules of the CD28/ B7 family. Immunol Rev. (2011) 241:180–205. doi: 10.1111/j.1600-065X.2011.01011.x, PMID: 21488898 PMC3077803

